# (5*S*,11a*S*)-5-Hydro­per­oxy-1,5,11,11a-tetra­hydro­[1]benzothieno[3,2-*f*]indol­izin-3(2*H*)-one

**DOI:** 10.1107/S1600536812045394

**Published:** 2012-11-10

**Authors:** Viktor Vrábel, Ľubomír Švorc, Štefan Marchalín, Peter Šafář

**Affiliations:** a Institute of Analytical Chemistry, Faculty of Chemical and Food Technology, Slovak Technical University, Radlinského 9, SK-812 37 Bratislava, Slovak Republic; b Institute of Organic Chemistry, Catalysis and Petrochemistry, Faculty of Chemical and Food Technology, Slovak Technical University, Radlinského 9, SK-812 37 Bratislava, Slovak Republic

## Abstract

The absolute configuration of the title compound, C_14_H_13_NO_3_S, was assigned from the synthesis and confirmed by the structure determination. The central six-membered ring of the indolizine moiety adopts an envelope conformation, with the greatest deviation from the mean plane of the ring being 0.661 (2) Å for the bridgehead C atom. The benzothiene ring attached to the indolizine ring system is planar to within 0.008 (2) Å. In the crystal, mol­ecules form chains parallel to the *b* axis *via* O—H⋯O hydrogen bonds.

## Related literature

For background to indolizines and their biological activity, see: Malonne *et al.* (1998 ▶); Medda *et al.* (2003 ▶); Sonnet *et al.* (2000 ▶); Campagna *et al.* (1990 ▶); Pearson & Guo (2001 ▶). For their synthesis, see: Šafář *et al.* (2009*a* ▶,*b* ▶). For compounds with similar properties, see: Švorc *et al.* (2008 ▶, 2009 ▶). For IR spectroscopy on similar compounds, see: Šafář *et al.* (2009*a* ▶). For conformational analysis, see: Nardelli (1983 ▶).
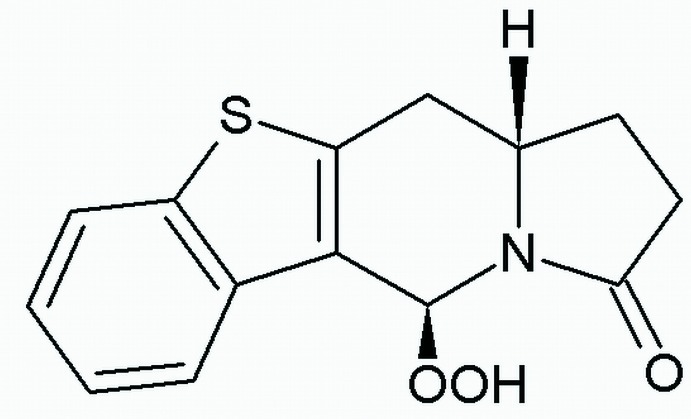


## Experimental

### 

#### Crystal data

C_14_H_13_NO_3_S
*M* *_r_* = 275.31
Monoclinic, 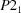

*a* = 7.8040 (5) Å
*b* = 7.9800 (4) Å
*c* = 10.2903 (5) Å
β = 99.458 (5)°
*V* = 632.13 (6) Å^3^
*Z* = 2
Mo *K*α radiation
μ = 0.26 mm^−1^
*T* = 295 K
0.25 × 0.20 × 0.15 mm

#### Data collection

Oxford Diffraction Xcalibur (Ruby, Gemi) diffractometer
Absorption correction: analytical [*CrysAlis RED* (Oxford Diffraction, 2009 ▶), based on expressions derived by Clark & Reid (1995 ▶)] *T*_min_ = 0.947, *T*_max_ = 0.974
21595 measured reflections
2555 independent reflections
2162 reflections with *I* > 2σ(*I*)
*R*_int_ = 0.028

#### Refinement

*R*[*F* ^2^ > 2σ(*F* ^2^)] = 0.027
*wR*(*F* ^2^) = 0.068
*S* = 1.00
2555 reflections
176 parameters
2 restraints
H atoms treated by a mixture of independent and constrained refinement
Δρ_max_ = 0.13 e Å^−3^
Δρ_min_ = −0.14 e Å^−3^
Absolute structure: Flack (1983 ▶), 1185 Friedel pairs
Flack parameter: −0.05 (6)

### 

Data collection: *CrysAlis CCD* (Oxford Diffraction, 2009 ▶); cell refinement: *CrysAlis CCD*; data reduction: *CrysAlis RED* (Oxford Diffraction, 2009 ▶); program(s) used to solve structure: *SHELXS97* (Sheldrick, 2008 ▶); program(s) used to refine structure: *SHELXL97* (Sheldrick, 2008 ▶); molecular graphics: *DIAMOND*, (Brandenburg, 2001 ▶), *PLATON* (Spek, 2009 ▶) and *WinGX* (Farrugia, 1999 ▶); software used to prepare material for publication: *SHELXL97*.

## Supplementary Material

Crystal structure: contains datablock(s) I, global. DOI: 10.1107/S1600536812045394/bg2481sup1.cif

Structure factors: contains datablock(s) I. DOI: 10.1107/S1600536812045394/bg2481Isup2.hkl

Supplementary material file. DOI: 10.1107/S1600536812045394/bg2481Isup3.cml

Additional supplementary materials:  crystallographic information; 3D view; checkCIF report

## Figures and Tables

**Table 1:** Hydrogen-bond geometry (Å, °)

*D*—H⋯*A*	*D*—H	H⋯*A*	*D*⋯*A*	*D*—H⋯*A*
O3—H3⋯O1^i^	0.84 (1)	1.86 (1)	2.6962 (19)	173 (2)

Symmetry code: (i) 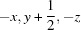.

**Fig. 1.**
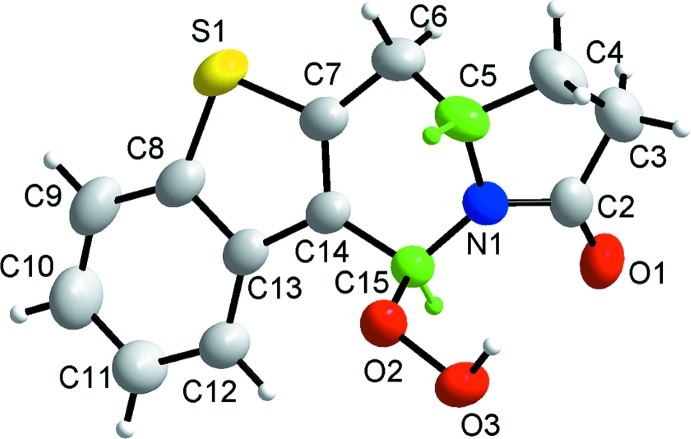
Molecular structure of the title compound with the atomic numbering scheme. Displacement ellipsoids are drawn at the 50% probability level (Brandenburg, 2001).

**Fig. 2.**
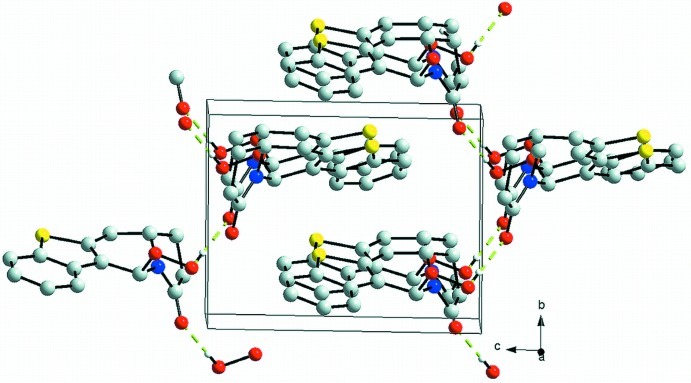
Packing view of the title compound. Molecular links are generated by O–H···O hydrogen bonds along the b axis which are shown by green dashed lines (Brandenburg, 2001).

## supplementary crystallographic information

### Comment

Heterocycles are involved in a wide range of biologically important chemical reactions in living organisms, and therefore they form one of the most important and well investigated classes of organic compounds. One group of heterocycles, indolizines, has received much scientific attention during the recent years. Indolizine derivatives have been found to possess a variety of biological activities such as antiinflammatory (Malonne *et al.*, 1998), antiviral (Medda *et al.*, 2003), aromatase inhibitory (Sonnet *et al.*, 2000), analgestic (Campagna *et al.*, 1990) and antitumor (Pearson & Guo, 2001) activities. As part of our recent efforts to synthesize novel polycyclic indolizine derivative, we report here the synthesis and molecular and crystal structure of the title compound, (I) (Fig. 1). The absolute configuration has been established without ambiguity from the anomalous dispersion of the S atom (Flack, 1983) and assigned consistent with the starting material. The expected stereochemistry of both atoms C5 and C15 was confirmed as S, see Fig. 1. The central N-heterocyclic ring is not planar and adopts a envelope conformation (Nardelli, 1983). A calculation of least-squares planes shows that this ring is puckered in such a manner that the five atoms C6, C7, C14, C15 and N1 are planar to within 0.075 (3) Å, while atom C5 is displaced from this plane with out-of-plane displacement of 0.661 (2) Å. The pyrrolidine ring is distorted towards a flat-envelope conformation, with atom C4 on the flap. Atom C4 is 0.402 (2) Å from the mean plane defined by atoms C5, N1, C2 and C3. The dihedral angle between the plane of the central N-heterocyclic ring and the plane of the pyrrolidine ring is 44.6 (1)°. Atom N1 is *sp*^2^-hybridized, as evidenced by the sum of the valence angles around it (358.1°). These data are consistent with conjugation of the lone-pair electrons on N1 with the adjacent carbonyl, similar to what is observed for amides. Intermolecular O—H···O hydrogen bonds link the molecules into infinite chains, which run parallel to the *b* axis (Fig. 2) and help to stabilize the crystal structure of the compound. The bond lengths of the carbonyl group C2=O1 is 1.233 (2) Å somewhat longer than typical carbonyl bonds. This may be due to the fact that atom O1 participates in intermolecular hydrogen bond.

### Experimental

To a solution of (11aS)-1,5,11,11*a*-tetrahydro[1]benzothieno[3,2-*f*] indolizin-3(2*H*)-one (0.041 mmol) in THF (2 ml) was added 3 drops of H_2_O_2_ at 0°C and the mixture was allowed to react at room temperature for 72 h. The colorless crystals were filtered off, and washing with dry n-hexane (1 ml) gave pure indolizinhydroperoxid.

### Refinement

All H atoms bonded to C were positioned with idealized geometry using a riding model with C–H = 0.93, 0.97 and 0.98 Å for aromatic, methylene and methine H, and constrained to ride on their parent atoms, with *U*_iso_(H) = 1.2*U*_eq_(C). The H3 atom on O3 was located in a difference map and finally refined isotropically with O—H distance fixed at 0.84 Å. The absolute configuration has been determined. The number of Friedel pairs is 1185.

### Crystal data

**Table:**

C_14_H_13_NO_3_S	*F*(000) = 288
*M**_r_* = 275.31	*D*_x_ = 1.446 Mg m^−^^3^
Monoclinic, *P*2_1_	Mo *K*α radiation, λ = 0.71073 Å
Hall symbol: P 2yb	Cell parameters from 10799 reflections
*a* = 7.8040 (5) Å	θ = 3.2–29.6°
*b* = 7.9800 (4) Å	µ = 0.26 mm^−^^1^
*c* = 10.2903 (5) Å	*T* = 295 K
β = 99.458 (5)°	Block, colourless
*V* = 632.13 (6) Å^3^	0.25 × 0.20 × 0.15 mm
*Z* = 2	

### Data collection

**Table:**

Oxford Diffraction Xcalibur (Ruby, Gemi) diffractometer	2555 independent reflections
Radiation source: fine-focus sealed tube	2162 reflections with *I* > 2σ(*I*)
Graphite monochromator	*R*_int_ = 0.028
Detector resolution: 10.4340 pixels mm^-1^	θ_max_ = 26.4°, θ_min_ = 4.4°
ω scans	*h* = −9→9
Absorption correction: analytical [*CrysAlis RED* (Oxford Diffraction, 2009), based on expressions derived by Clark & Reid (1995)]	*k* = −9→9
*T*_min_ = 0.947, *T*_max_ = 0.974	*l* = −12→12
21595 measured reflections	

### Refinement

**Table:**

Refinement on *F*^2^	Secondary atom site location: difference Fourier map
Least-squares matrix: full	Hydrogen site location: inferred from neighbouring sites
*R*[*F*^2^ > 2σ(*F*^2^)] = 0.027	H atoms treated by a mixture of independent and constrained refinement
*wR*(*F*^2^) = 0.068	*w* = 1/[σ^2^(*F*_o_^2^) + (0.0441*P*)^2^] where *P* = (*F*_o_^2^ + 2*F*_c_^2^)/3
*S* = 1.00	(Δ/σ)_max_ < 0.001
2555 reflections	Δρ_max_ = 0.13 e Å^−^^3^
176 parameters	Δρ_min_ = −0.14 e Å^−^^3^
2 restraints	Absolute structure: Flack (1983), 1185 Friedel pairs
Primary atom site location: structure-invariant direct methods	Flack parameter: −0.05 (6)

### Special details

**Table:**

Geometry. All e.s.d.'s (except the e.s.d. in the dihedral angle between two l.s. planes) are estimated using the full covariance matrix. The cell e.s.d.'s are taken into account individually in the estimation of e.s.d.'s in distances, angles and torsion angles; correlations between e.s.d.'s in cell parameters are only used when they are defined by crystal symmetry. An approximate (isotropic) treatment of cell e.s.d.'s is used for estimating e.s.d.'s involving l.s. planes.
Refinement. Refinement of *F*^2^ against ALL reflections. The weighted *R*-factor *wR* and goodness of fit *S* are based on *F*^2^, conventional *R*-factors *R* are based on *F*, with *F* set to zero for negative *F*^2^. The threshold expression of *F*^2^ > σ(*F*^2^) is used only for calculating *R*-factors(gt) *etc*. and is not relevant to the choice of reflections for refinement. *R*-factors based on *F*^2^ are statistically about twice as large as those based on *F*, and *R*- factors based on ALL data will be even larger.

### Fractional atomic coordinates and isotropic or equivalent isotropic displacement parameters (Å^2^)

**Table:**

	*x*	*y*	*z*	*U*_iso_*/*U*_eq_	
C2	−0.1152 (2)	0.0934 (2)	0.10939 (14)	0.0487 (4)	
C3	−0.2994 (2)	0.1556 (3)	0.07868 (18)	0.0625 (5)	
H3B	−0.3714	0.1014	0.1347	0.075*	
H3A	−0.3483	0.1340	−0.0127	0.075*	
C4	−0.2872 (2)	0.3432 (3)	0.1057 (2)	0.0662 (5)	
H4B	−0.3852	0.3817	0.1447	0.079*	
H4A	−0.2838	0.4054	0.0252	0.079*	
C5	−0.1161 (2)	0.3639 (3)	0.20284 (16)	0.0527 (4)	
H5	−0.0563	0.4666	0.1834	0.063*	
C6	−0.1345 (2)	0.3584 (3)	0.34751 (18)	0.0584 (4)	
H6B	−0.1750	0.4661	0.3741	0.070*	
H6A	−0.2190	0.2739	0.3613	0.070*	
C7	0.0371 (2)	0.31816 (19)	0.42819 (16)	0.0490 (4)	
C8	0.2927 (2)	0.2798 (2)	0.60665 (15)	0.0497 (4)	
C9	0.4223 (3)	0.2701 (3)	0.71705 (16)	0.0633 (5)	
H9	0.4012	0.3056	0.7990	0.076*	
C10	0.5810 (3)	0.2073 (3)	0.70255 (19)	0.0707 (6)	
H10	0.6691	0.2028	0.7752	0.085*	
C11	0.6131 (3)	0.1499 (3)	0.58133 (19)	0.0634 (5)	
H11	0.7218	0.1064	0.5743	0.076*	
C12	0.4862 (2)	0.1569 (2)	0.47181 (16)	0.0511 (4)	
H12	0.5084	0.1174	0.3912	0.061*	
C13	0.3233 (2)	0.22356 (19)	0.48218 (15)	0.0428 (4)	
C14	0.17251 (19)	0.24634 (19)	0.38248 (14)	0.0412 (4)	
C15	0.15873 (19)	0.1929 (2)	0.24080 (14)	0.0413 (4)	
H15	0.1927	0.0751	0.2363	0.050*	
N1	−0.01704 (17)	0.21547 (16)	0.17396 (12)	0.0447 (3)	
O1	−0.06189 (17)	−0.04709 (17)	0.08604 (12)	0.0627 (3)	
O2	0.27750 (15)	0.29669 (15)	0.18624 (11)	0.0513 (3)	
O3	0.28192 (17)	0.23328 (18)	0.05339 (11)	0.0597 (3)	
H3	0.207 (2)	0.299 (3)	0.013 (2)	0.098 (9)*	
S1	0.08315 (6)	0.35880 (6)	0.59656 (4)	0.06228 (15)	

### Atomic displacement parameters (Å^2^)

**Table:**

	*U*^11^	*U*^22^	*U*^33^	*U*^12^	*U*^13^	*U*^23^
C2	0.0512 (10)	0.0610 (11)	0.0332 (7)	−0.0060 (9)	0.0043 (7)	0.0038 (8)
C3	0.0518 (11)	0.0782 (14)	0.0546 (10)	−0.0038 (10)	0.0001 (8)	0.0057 (9)
C4	0.0501 (10)	0.0692 (13)	0.0765 (12)	0.0052 (11)	0.0018 (9)	0.0209 (11)
C5	0.0440 (9)	0.0452 (9)	0.0699 (10)	0.0038 (9)	0.0121 (8)	0.0093 (9)
C6	0.0508 (9)	0.0560 (9)	0.0702 (10)	0.0095 (10)	0.0151 (8)	−0.0066 (10)
C7	0.0520 (10)	0.0446 (10)	0.0529 (9)	−0.0003 (8)	0.0156 (8)	−0.0048 (7)
C8	0.0679 (11)	0.0400 (8)	0.0435 (9)	0.0012 (8)	0.0161 (8)	0.0007 (7)
C9	0.0933 (15)	0.0563 (11)	0.0402 (9)	0.0050 (11)	0.0102 (9)	0.0006 (8)
C10	0.0872 (16)	0.0703 (14)	0.0492 (10)	0.0112 (12)	−0.0050 (10)	0.0097 (9)
C11	0.0673 (12)	0.0648 (12)	0.0560 (10)	0.0171 (10)	0.0042 (9)	0.0099 (9)
C12	0.0589 (11)	0.0514 (10)	0.0439 (9)	0.0086 (9)	0.0111 (8)	0.0041 (7)
C13	0.0549 (9)	0.0338 (8)	0.0411 (7)	−0.0015 (7)	0.0120 (7)	0.0016 (6)
C14	0.0467 (9)	0.0354 (8)	0.0433 (8)	−0.0009 (7)	0.0125 (7)	−0.0009 (6)
C15	0.0405 (8)	0.0433 (9)	0.0408 (8)	−0.0002 (7)	0.0084 (6)	0.0009 (6)
N1	0.0429 (7)	0.0457 (7)	0.0458 (7)	0.0001 (6)	0.0082 (6)	0.0021 (6)
O1	0.0674 (9)	0.0619 (8)	0.0554 (7)	−0.0007 (7)	0.0000 (6)	−0.0159 (6)
O2	0.0498 (6)	0.0609 (7)	0.0445 (6)	−0.0061 (5)	0.0121 (5)	0.0000 (5)
O3	0.0607 (8)	0.0756 (9)	0.0452 (7)	0.0118 (7)	0.0163 (6)	0.0007 (6)
S1	0.0745 (3)	0.0630 (3)	0.0537 (2)	0.0086 (3)	0.0234 (2)	−0.0118 (2)

### Geometric parameters (Å, º)

**Table:**

C2—O1	1.233 (2)	C8—C13	1.414 (2)
C2—N1	1.345 (2)	C8—S1	1.7400 (19)
C2—C3	1.505 (3)	C9—C10	1.366 (3)
C3—C4	1.523 (3)	C9—H9	0.9300
C3—H3B	0.9700	C10—C11	1.390 (3)
C3—H3A	0.9700	C10—H10	0.9300
C4—C5	1.539 (3)	C11—C12	1.374 (3)
C4—H4B	0.9700	C11—H11	0.9300
C4—H4A	0.9700	C12—C13	1.398 (2)
C5—N1	1.471 (2)	C12—H12	0.9300
C5—C6	1.519 (2)	C13—C14	1.440 (2)
C5—H5	0.9800	C14—C15	1.506 (2)
C6—C7	1.490 (2)	C15—O2	1.4252 (19)
C6—H6B	0.9700	C15—N1	1.441 (2)
C6—H6A	0.9700	C15—H15	0.9800
C7—C14	1.353 (2)	O2—O3	1.4634 (16)
C7—S1	1.7411 (17)	O3—H3	0.841 (2)
C8—C9	1.393 (3)		
			
O1—C2—N1	124.86 (17)	C13—C8—S1	110.95 (13)
O1—C2—C3	126.89 (16)	C10—C9—C8	118.72 (16)
N1—C2—C3	108.17 (16)	C10—C9—H9	120.6
C2—C3—C4	104.88 (16)	C8—C9—H9	120.6
C2—C3—H3B	110.8	C9—C10—C11	121.29 (18)
C4—C3—H3B	110.8	C9—C10—H10	119.4
C2—C3—H3A	110.8	C11—C10—H10	119.4
C4—C3—H3A	110.8	C12—C11—C10	120.70 (18)
H3B—C3—H3A	108.8	C12—C11—H11	119.7
C3—C4—C5	104.22 (16)	C10—C11—H11	119.7
C3—C4—H4B	110.9	C11—C12—C13	119.67 (15)
C5—C4—H4B	110.9	C11—C12—H12	120.2
C3—C4—H4A	110.9	C13—C12—H12	120.2
C5—C4—H4A	110.9	C12—C13—C8	118.72 (15)
H4B—C4—H4A	108.9	C12—C13—C14	129.82 (14)
N1—C5—C6	108.21 (14)	C8—C13—C14	111.46 (14)
N1—C5—C4	102.19 (16)	C7—C14—C13	113.53 (14)
C6—C5—C4	114.92 (14)	C7—C14—C15	121.41 (14)
N1—C5—H5	110.4	C13—C14—C15	125.04 (13)
C6—C5—H5	110.4	O2—C15—N1	111.66 (12)
C4—C5—H5	110.4	O2—C15—C14	105.62 (12)
C7—C6—C5	109.33 (13)	N1—C15—C14	109.74 (12)
C7—C6—H6B	109.8	O2—C15—H15	109.9
C5—C6—H6B	109.8	N1—C15—H15	109.9
C7—C6—H6A	109.8	C14—C15—H15	109.9
C5—C6—H6A	109.8	C2—N1—C15	124.42 (15)
H6B—C6—H6A	108.3	C2—N1—C5	114.03 (14)
C14—C7—C6	125.42 (15)	C15—N1—C5	119.65 (13)
C14—C7—S1	112.32 (13)	C15—O2—O3	106.49 (11)
C6—C7—S1	122.26 (12)	O2—O3—H3	97.2 (18)
C9—C8—C13	120.88 (17)	C8—S1—C7	91.72 (7)
C9—C8—S1	128.16 (13)		

### Hydrogen-bond geometry (Å, º)

**Table:**

*D*—H···*A*	*D*—H	H···*A*	*D*···*A*	*D*—H···*A*
O3—H3···O1^i^	0.84 (1)	1.86 (1)	2.6962 (19)	173 (2)

Symmetry code: (i) −*x*, *y*+1/2, −*z*.
